# Oxytocin and social salience: a call for gene-environment interaction research

**DOI:** 10.3389/fnins.2013.00199

**Published:** 2013-10-31

**Authors:** Benjamin A. Tabak

**Affiliations:** Department of Psychology, University of CaliforniaLos Angeles, Los Angeles, CA, USA

**Keywords:** oxytocin, oxytocin receptor gene, OXTR, gene-environment interaction, genetic association study

The role of the neuropeptide oxytocin in social cognition and prosocial behavior continues to fascinate the scientific community (Bartz et al., 2011b). However, as our knowledge of oxytocin's influence on social processes increases, a more complex picture continues to emerge. Studies have begun to identify individual differences and environmental contexts that substantially alter the effects of oxytocin administration (Bartz et al., 2011b; Macdonald, 2012). In addition, interpersonal distress has been positively correlated with plasma oxytocin (e.g., Tabak et al., 2011), and evidence for potential negative consequences of oxytocin administration continues to accumulate (Miller, 2013)—calling into question the view that oxytocin represents a social panacea (Pfeiffer, 2013). Although questions remain about how to properly measure oxytocin (Szeto et al., 2011; McCullough et al., 2013), and whether intranasal administration of oxytocin crosses the blood-brain barrier (Churchland and Winkielman, 2012; but see, Neumann et al., 2013), a developing theme in human oxytocin research is that early environmental experiences and/or attachment styles appear to moderate the effects of oxytocin administration (Bartz et al., 2010, 2011a; van IJzendoorn et al., 2011; De Dreu, 2012a; Riem et al., 2013).

Translational research has demonstrated that early environmental experiences can alter the oxytocin system (Champagne et al., 2001; Bales and Perkeybile, 2012), which may have long-lasting effects that impact adult human functioning (e.g., Heim et al., 2009; Strathearn et al., 2009; Feldman et al., 2013). Based on findings such as these, an increasing number of studies have begun to investigate how variation on the gene encoding for the oxytocin receptor (OXTR) may relate to socially relevant normative and pathological phenotypes. To date, the most commonly studied marker on OXTR is rs53576, an adenine-to-guanine single nucleotide polymorphism (SNP) that lies in the third intronic region. Although most published studies have found associations with the rs53576 G allele and beneficial aspects of social cognition and behavior (e.g., Saphire-Bernstein et al., 2011; Krueger et al., 2012), several studies have found no relationship between rs53576 genotype and similar social phenotypes (e.g., Apicella et al., 2010; Cornelis et al., 2012; Tabak et al., 2013), or opposite relationships (i.e., where the A allele is protective; Costa et al., 2009). Although some of these discrepancies likely result from known issues in genetic association studies (e.g., Ioannidis et al., 2001; Lin et al., 2007), findings from studies of endogenous oxytocin (Strathearn et al., 2009), oxytocin administration (van IJzendoorn et al., 2011), and OXTR (McQuaid et al., 2013) suggest that one method through which studies might yield more consistent findings is to include measures of environmental experiences in order to examine gene-environment interactions.

In a study recently published in this journal, McQuaid et al. (2013) examined how rs53576 genotype moderated the relationship between childhood maltreatment and depression symptoms in a racially/ethnically diverse sample. The authors found an association between individuals who carried the G allele on rs53576 and increased depression symptomology compared to those with the AA genotype. Furthermore, the authors discovered a meditational role of distrust/cynicism, suggesting that depression symptoms resulted (or co-occurred) in part from developing a distrustful attitude toward others after having suffered childhood maltreatment. McQuaid et al.'s 2013 findings are in agreement with Bradley et al. (2011) who studied a larger sample (*n* = 595) of 18–90 year old African Americans with low-incomes: they found that individuals with the GG genotype on rs53576 who experienced severe childhood maltreatment were more likely to have disorganized attachment styles and higher levels of emotional dysregulation.

Thus, in contrast to the majority of published studies focusing on rs53576, Bradley et al. (2011) and McQuaid et al. (2013) found that it was the G allele that represented risk rather than protection when examined in the context of childhood maltreatment. Initially, these findings seem to contradict those from previous studies; however, these results are in agreement with the “social salience” hypothesis of oxytocin, which proposes that oxytocin increases the salience of both positive and negative social cues (Shamay-Tsoory et al., 2009). Following this logic, enhanced social salience may improve social cognition and increase prosocial behavior for individuals in positive environments, but have detrimental effects for others in negative environments (Bartz et al., 2011b; Bakermans-Kranenburg and van IJzendoorn, 2013), perhaps because these environments encourage the view that others are untrustworthy (Bakermans-Kranenburg et al., 2012). This hypothesis has gained some traction in light of recent findings demonstrating that oxytocin does not promote (or reduces) prosocial tendencies toward anonymous others or out-group members (Declerck et al., 2010; De Dreu et al., 2010; Radke and de Bruijn, 2012). However, the ways in which oxytocin administration may differentially affect people depending on attachment anxiety and/or avoidance continue to be explored (Bartz, 2012; De Dreu, 2012b).

Findings from Bradley et al. (2011) and McQuaid et al. (2013) involving rs53576 are also in agreement with the differential susceptibility hypothesis (Belsky et al., 2009), which posits that certain genetic variants can represent risk or protection depending upon whether or not the environment is positive or negative (for a discussion of differential susceptibility as related to another OXTR SNP, rs2254298, see Brüne, 2012). Importantly, the function of rs53576 is unknown. In addition, results from McQuaid et al. (2013) were based on a cross-sectional design, retrospective self-reports, and a small heterogeneous sample, which increases the potential for false-positive findings (Moffitt et al., 2006). As a result, the authors appropriately note that replication with larger samples and more sophisticated methodology is needed. Nonetheless, results from McQuaid et al. (2013) are in agreement with studies demonstrating the plasticity of the oxytocin system in both animals and humans and suggest that it is in the best interest of researchers investigating genetic associations (as well as epigenetic mechanisms; Kumsta et al., 2013) in oxytocin system relevant genes to measure both positive and negative environmental experiences for the purpose of examining gene-environment interactions.
